# Efficacy and safety of PARP inhibitor maintenance therapy in elderly ovarian cancer patients: a real-world single-center retrospective cohort study

**DOI:** 10.3389/fonc.2026.1737880

**Published:** 2026-06-08

**Authors:** Jingjing Lu, Xuelian Song, Chen Zhang, Yi Zhu, Huarui Yang, Yi Li

**Affiliations:** 1 Department of Obstetrics and Gynecology, Peking University People’s Hospital, Beijing, China; 2 Department of Obstetrics and Gynecology, Handan Central Hospital, Handan, Hebei, China

**Keywords:** maintenance therapy, older adult patients, ovarian cancer, PARP inhibitors, real-world study

## Abstract

**Background:**

PARP(Poly(ADP-ribose) polymerase) inhibitors are established as effective treatment for ovarian cancer; however, there have been no systematic studies of PARP inhibitor use in older patients with ovarian cancer.

**Methods:**

This study was conducted at Peking University People’s Hospital. Archival data from patients with advanced age (≥ 65 years) with ovarian cancer between March 2018 and September 2023, who received olaparib or niraparib as maintenance therapy following either first-line platinum-based chemotherapy or platinum-sensitive recurrence (PSR) chemotherapy and achieved complete (CR) or partial response (PR). The two clinical scenarios (first-line maintenance and PSR maintenance) were analyzed separately.

**Results:**

Of 56 included patients, 20 (35.7%) and 34 (60.7%) received olaparib and niraparib, respectively, while 2 (3.6%) switched from olaparib to niraparib due to intolerance. The mean age was 70.14 ± 4.63 years. Median progression-free survival (mPFS) was 24 months in 35 (62.5%) patients receiving PARP inhibitors as first-line maintenance therapy(1L-group). In the 1L group, CA125 level, whether CR was reached, *BRCA* mutation status, and R0 at initial surgery before PARP inhibitor therapy were associated with PFS in univariate analysis, and the latter three factors were independently associated with PFS on multivariate analysis. In the PSR group, among patients with PSR ≥12 months (n=13), mPFS was not reached; among those with PSR 6–12 months (n=7), mPFS was 9.6 months. Anemia (22.7%) was the most frequent grade 3–4 adverse event in the olaparib group, whereas thrombocytopenia (11.1%) was more common in the niraparib group. Both groups of patients experienced dose reduction, interruption, and discontinuation within the first 6 months. Niraparib had a lower termination rate than olaparib, and long-term PARP inhibitor use was generally well-tolerated.

**Conclusions:**

PARP inhibitor (olaparib and niraparib) maintenance therapy yields favorable clinical outcomes for elderly ovarian cancer patients. Three clinical factors, attainment R0(no residual disease) after initial surgery, BRCA mutation, and CA-125 ≤10 U/mL before PARP inhibitor treatment, were predictive of longer PFS in elderly ovarian cancer patients undergoing first-line maintenance therapy with PARP inhibitors.

## Introduction

Ovarian cancer is the most lethal gynecological malignancy. In 2020, there were 313,959 new cases and 207,252 fatalities worldwide (1). Almost all patients with advanced ovarian cancer relapse, with a median time to recurrence of 15 months from diagnosis (2). With a gradual reduction in disease-free intervals as treatment lines progress, the resistance to platinum-based therapies develops (3).

Recently, clinical studies of patients with ovarian cancer have demonstrated remarkable efficacy of PARP inhibitors, a category of anticancer medications that targeting DNA repair mechanisms. SOLO-1 study have demonstrated that olaparib as first-line maintenance treatment for newly-diagnosed advanced ovarian cancer patients with *BRCA* gene mutation significantly prolonged PFS (4). Additionally, PRIMA and PRIME study indicate the benefits of niraparib as maintenance therapy across the entire spectrum of ovarian cancer patients who response to first-line chemotherapy, irrespective of biomarker status (5, 6). Further, PARP inhibitors have demonstrated efficacy in extending PFS in patients with platinum-sensitive recurrent ovarian cancer (PSROC) in trials such as SOLO 2 (7), NOVA (8), NORA (9), and others (10).

Strict inclusion and exclusion criteria are used in RCTs to minimize bias and the results are frequently hard to duplicate in real-world settings (11). Unfortunately, few Chinese or Asian people have participated in prior worldwide clinical studies. In clinical studies of PARP inhibitor maintenance therapy, including SOLO1 (4), PRIMA (5), SOLO2 (7), and NOVA (8), the median age of patients was generally 50–60 years. In the PRIME (6) trial, the median (range) age of the Chinese population receiving niraparib (n = 255) was 53 (32–77) years. Population aging is expected to be a significant social trend worldwide (12). In China, the proportion of people aged ≥60 years is predicted to exceed 30% by 2035 (13). An early large-scale study based on the U.S. Surveillance, Epidemiology, and End Results (SEER) Program database found that patients aged 65 years and older accounted for approximately 42% of all ovarian cancer patients. More importantly, elderly women were more likely to be at an advanced stage at initial diagnosis (14). The European OVCAD study also confirmed that the proportion of patients aged ≥70 years with an Eastern Cooperative Oncology Group (ECOG) performance status score of ≥2 was significantly higher than that of patients aged <70 years (10.6% vs 3.1%, p=0.047) (15). Elderly patients have a heavier burden of comorbidities (16). The proportion of elderly patients receiving standard treatment (cytoreductive surgery combined with platinum-based chemotherapy) is significantly low (17). However, geriatric assessment tools that could guide treatment individualization are not routinely incorporated in clinical practice, and the distinct features of elderly patients—particularly their higher comorbidity burden and altered drug tolerance—have not been systematically examined in the context of PARP inhibitor maintenance therapy.

Therefore, we conducted this study to evaluate the efficacy, safety, and factors associated with long-term benefits and tolerability of PARP inhibitor therapy in elderly patients with advanced ovarian cancer, separately for those receiving first-line maintenance therapy and those receiving maintenance after platinum-sensitive recurrent disease.

## Methods

### Patient population

In this study, “elderly” was defined as age ≥65 years, consistent with the National Comprehensive Cancer Network (NCCN) Geriatric Oncology Guidelines (18) and the Chinese Expert Consensus on elderly ovarian cancer (19). This study was conducted at the People’s Hospital of Peking University. The study was approved by the Ethics Committee, and included women diagnosed with invasive ovarian epithelial cancer, fallopian tube cancer, or primary peritoneal cancer (collectively referred to as ovarian cancer) who had achieved complete response (CR) or partial response (PR) to their last platinum-based chemotherapy and were treated with PARP inhibitor (olaparib or niraparib) as maintenance therapy. Patients were included in one of two distinct clinical scenarios: (1) first-line maintenance therapy, defined as maintenance following a response to first-line platinum-based chemotherapy; (2) maintenance after platinum-sensitive recurrence (PSR) disease, defined as maintenance following a response to subsequent platinum-based chemotherapy for recurrent disease with a PSR of ≥6 months. Archival data for consecutive patients were collected from March 2018 to September 2023. The two cohorts were analyzed separately throughout the study.

PFS, defined as the time (in months) from initiation of PARP inhibitors to disease progression (based on Response Evaluation Criteria in Solid Tumors (RECIST) 1.1 (20) and Gynecologic Cancer Intergroup (GCIG) CA-125 criteria (21)), end of follow-up, or death from any cause, was used to evaluate the effectiveness of the therapy. PFS was calculated independently for the first-line maintenance cohort and the PSR maintenance cohort. Adverse events(AEs) were categorized using the Common Terminology Criteria for Adverse Events (CTCAE) version 5.0.

**Figure 1:**
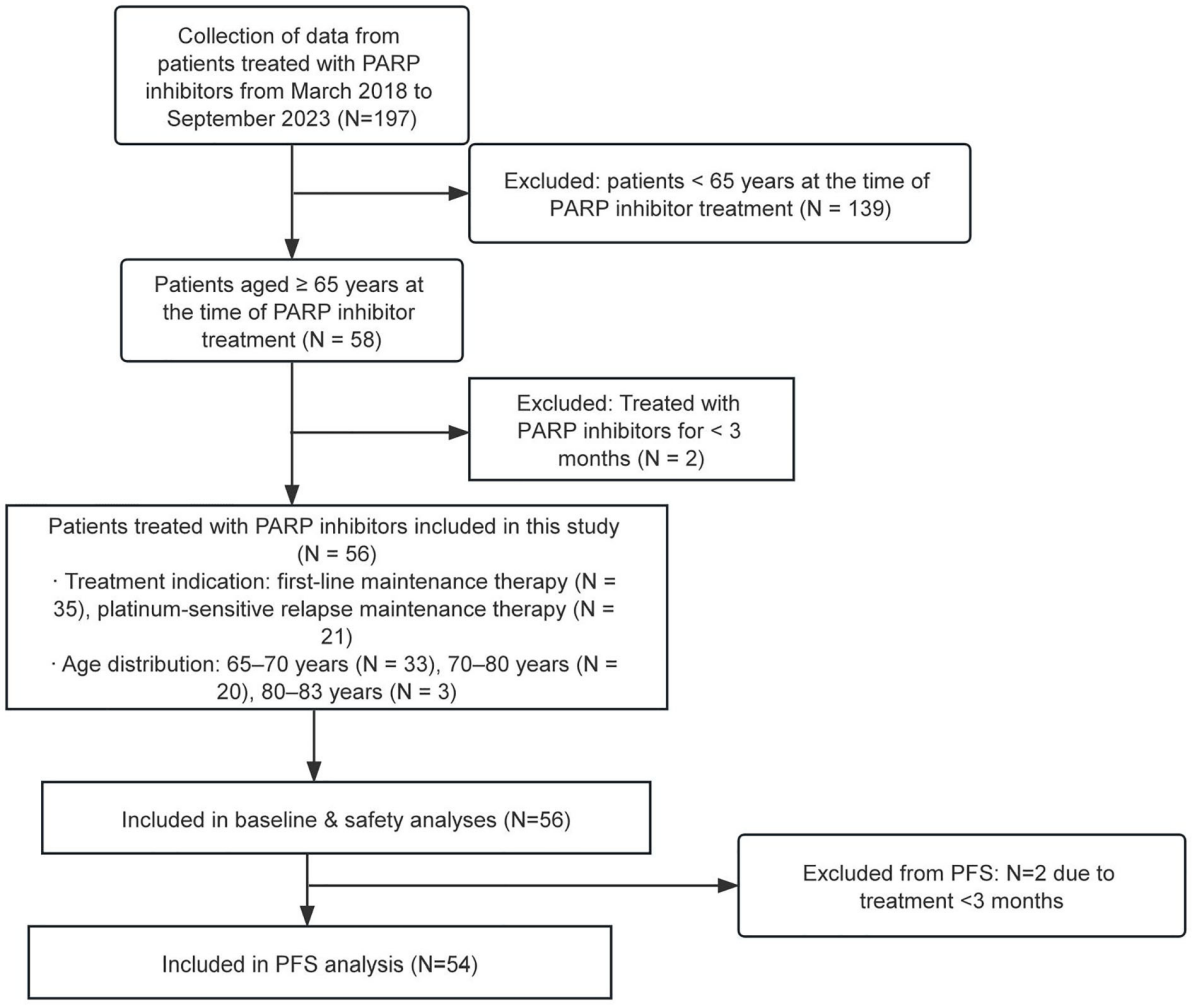
Study Enrollment flow diagram. Abbreviations: PARP, poly(ADP-ribose) polymerase.

For baseline characteristics and safety analyses, all 56 patients who received at least one dose of PARP inhibitor were included. For progression-free survival (PFS) analysis, patients who discontinued treatment within 3 months (n=2) were excluded because they did not undergo response assessment, leaving 54 patients for survival analysis (**Figure 1**).

### Data collection

Clinical information, including patient demographics, clinicopathological characteristics, BRCA mutation status, residual status after primary tumor cytoreduction, secondary cytoreduction, and recurrence, etc., were collected from medical records. BRCA mutation was defined as the presence of any deleterious or suspected deleterious mutation based on blood or tumor testing, while others were categorized as BRCA wild-type. The incidence of AEs was recorded, as well as dose reductions, dose interruptions, and discontinuations due to AEs. Missing information was supplemented through telephone follow-up or face-to-face questioning.

The initial oral dose of olaparib was 300 mg twice a day, and was maintained for at least 24 months, or until disease progression if toxicity was tolerated. Based on the results of the retrospective RADAR analysis in the NOVA study (22) and the fact that almost all patients with ovarian cancer in China have a body weight <77 kg, all patients were started on maintenance therapy with niraparib at an individual starting dose of 200 mg once daily in routine practice at our institution and continued for at least 36 months, or until disease progression if toxicity was under control. Patients were recommended to attend clinic visits at least once a month for prescriptions, symptom assessment, and laboratory tests (including complete blood count and CA-125 at minimum) and every 3–6 months for tumor evaluation (primarily through imaging scans, mainly computed tomography scan) until objective disease progression or intolerable toxicity occurred.

### Statistical analysis

Continuous variables are reported as mean and standard deviation. Comparisons between groups were made using the t-test. Count data are expressed as frequencies and rates (%), and comparisons between groups were made using the chi-square or Fisher’s exact test. All descriptive and comparative analyses were performed separately for the first-line maintenance cohort and the PSR maintenance cohort; no direct statistical comparison was made between the two cohorts due to inherent differences in disease status and treatment goals. The Kaplan-Meier method was used to estimate the median PFS (mPFS) and examine the impact of different clinical characteristics on the effectiveness of PARP inhibitors within each cohort. For multivariate analyses, Cox proportional hazard regression analyses performed to calculate hazard ratios (HRs) and 95% confidence intervals (CIs). Missing data were minimal (<5% for any variable). Therefore, complete case analysis was used, and no imputation was performed. IBM SPSS statistics software (version 23.0) was used for statistical analysis. A P-value of <0.05 was considered statistically significant.

## Results

### Patient disposition and baseline characteristics

56 women were ultimately included in the cohort, based on the inclusion and exclusion criteria (**Figure 1**). Out of the total 20 (35.7%) patients received olaparib, while 34 (60.7%) received niraparib. Two patients (3.6%) who switched from olaparib to niraparib due to intolerance (treatment duration <3 months) were analyzed in the niraparib group.

Baseline clinical and demographic characteristics are summarized in **Table 1**. The mean age at PARP inhibitor initiation was 70.14 ± 4.63 years. 35 patients (62.5%) received first-line maintenance, 13 patients (23.2%) received maintenance after a platinum-sensitive recurrence interval ≥12 months, and 8 (14.3%) after an interval of 6–12 months. A known BRCA mutation was present in 13 patients (23.2%), with a significantly higher proportion in the olaparib group (55.0% vs. 5.6%, p<0.001).Detailed comparisons of other baseline variables between treatment groups are shown in **Table 1**.

**Table 1:** Clinical characteristics of patients with ovarian cancer included in this study.

	Number of cases N = 56	Olaparib N = 20	Niraparib N = 36	P
Age, years	70.14±4.63^#^	70.40±5.46^#^	70.00±4.18^#^	0.379
65–70	33 (58.9%)	12 (60%)	21 (58.3%)	
70–80	20 (35.7%)	6 (30%)	14 (38.9%)	
80–83	3 (5.4%)	2 (10%)	1 (2.8%)	
BSA, /m^2^	1.63±0.14^#^	1.63±0.17^#^	1.63±0.12^#^	0.993
BMI, kg/m^2^	23.52±3.40^#^	24.22±4.49^#^	23.13±2.60^#^	0.255
Weight, kg	59.32±9.44^#^	60.28±12.51^#^	59.32±9.44^#^	0.578
Complication				
Yes	54 (96.4%)	20 (100.0%)	34 (94.4%)	0.532
No	2 (3.6%)	0 (0.0%)	2(5.6%)	
Indications for receiving PARP inhibitors				
First-line maintenance	35 (62.5%)	9 (45.0%)	26 (72.2%)	0.107
PSR (≥ 12 months)	13 (23.2%)	6 (30.0%)	7 (19.4%)	
PSR (6–12 months)	8 (14.3%)	5 (25.0%)	3 (8.3%)	
*BRCA* mutation status				
Mutant type	13 (23.2%)	11 (55.0%)	2 (5.6%)	0.000*
Wild-type	38 (67.9%)	6 (30.0%)	32 (88.9%)	
Unknown	5 (8.9%)	3 (15.0%)	2 (5.6%)	
Family history of ovarian or breast cancer				
Yes	7 (12.5%)	4 (20.0%)	3 (8.3%)	0.234
No	49 (87.5%)	16 (80.0%)	33 (91.7%)	
Family history of malignancy other than ovarian/breast				
Yes	6 (10.7%)	4 (20.0%)	2 (5.6%)	0.172
No	50 (89.3%)	16 (80.0%)	34 (94.4%)	
Personal history of breast cancer				
Yes	5 (8.9%)	4 (20.0%)	1 (2.8%)	0.050*
No	51 (91.1%)	16 (80.0%)	32 (97.2%)	
FIGO stage at initial diagnosis				
I	3 (5.4%)	0 (0.0%)	3 (8.3%)	0.822
II	3 (5.4%)	1 (5.0%)	2 (5.6%)	
III	40 (71.4%)	15 (75.0%)	25 (69.4%)	
IV	10 (17.9%)	4 (20.0%)	6 (16.7%)	
Primary site				
Ovaries	45 (80.4%)	15 (75.0%)	30 (83.3%)	0.294
Fallopian tube	6 (10.7%)	4 (20.0%)	2 (5.6%)	
Peritoneum	5 (8.9%)	1 (5.0%)	4 (11.1%)	
Histology				
High-grade plasmacytoid carcinoma	50 (89.3%)	19 (95.0%)	31 (86.1%)	0.770
Endometrioid/clear cell carcinoma	5 (8.9%)	1 (5.0%)	4 (11.1%)	
Plasmacytoid mucinous tumor	1 (1.8%)	0 (0.0%)	1 (2.8%)	
Neoadjuvant chemotherapy				
Yes	16 (28.6%)	3 (15.0%)	13 (36.1%)	0.127
No	40 (71.4%)	17 (85.0%)	23 (63.9%)	
Primary tumor cytoreduction				
R0	28 (50.0%)	12 (60.0%)	16 (44.4%)	0.146
R1	17 (30.4%)	6 (30.0%)	11 (30.6%)	
R2	7 (12.5%)	0 (0.0%)	7 (19.4%)	
inoperable	4 (7.1%)	2 (10.0%)	2 (5.6%)	
Secondary cytoreduction				
Yes	12 (21.4%)	6 (30.0%)	6 (16.7%)	0.313
No	44 (78.6%)	14 (70.0%)	30 (83.3%)	
Previous platinum-based chemotherapy				
≤1 line	37 (66.1%)	10 (50.0%)	27 (75.0%)	0.173
2 lines	12 (21.4%)	6 (30.0%)	6 (16.7%)	
≥3 lines	7 (12.5%)	4 (20.0%)	3 (8.3%)	
Last chemotherapy was combined with bevacizumab				
Yes	4 (7.1%)	1 (5.0%)	3 (8.3%)	1.000
No	52 (92.9%)	19 (95.0%)	33 (91.7%)	
Time from last chemotherapy to initiation of PARP inhibitors				
≤4 weeks	9 (16.1%)	3 (15.0%)	6 (16.7%)	0.568
4–8 weeks	24 (42.9%)	7 (35.0%)	17 (47.2%)	
≥8 weeks	23 (41.1%)	10 (50.0%)	13 (36.1%)	
CA-125 level before receiving PARP inhibitors ( (U/ML))				
≤10	20 (35.7%)	6 (30.0%)	14 (38.9%)	0.666
10–35	25 (44.6%)	9 (45.0%)	16 (44.4%)	
≥35	11 (19.6%)	5 (25.0%)	6 (16.7%)	
Objective response to the last platinum-based therapy				
CR	30 (53.6%)	10 (50.0%)	20 (55.6%)	0.783
PR	26 (46.4%)	10 (50.0%)	16 (44.4%)	

#Mean ± standard deviation. *Significant difference.

PARP, poly(ADP-ribose) polymerase. PSR, platinum-sensitive recurrence. CR, complete response. PR, partial (PR) response; BMI ,body mass index; BSA ,body surface area;

FIGO, International Federation of Gynecology and Obstetrics; R0,no residual disease; R1, residual ≤1 cm; R2,residual >1 cm.

**Statistical analysis:** Comparisons between Continuous variables’ groups were made using the t-test. Comparisons between Count variables’ groups were made using the chi-square or Fisher’s exact test. For multivariate analyses, Cox proportional hazard regression analyses performed to calculate hazard ratios (HRs) and 95% confidence intervals (CIs).

### Efficacy outcomes

The median follow-up time for the entire cohort was 28.0months(range: 3-63months). For survival analysis, 2 of the 56 patients who discontinued treatment within 3 months were excluded, leaving 54 patients for PFS analysis (**Table 2**). In the first-line maintenance group (n=34), median progression-free survival (mPFS) was 24.0 months. In the PSR group, mPFS was not reached for patients with a treatment-free interval ≥12 months (n=13) and was 9.6 months for those with an interval of 6–12 months (n=7). Given the limited sample size of the PSR group, subsequent analyses focused on patients receiving first−line maintenance therapy.

**Table 2:** mPFS of ovarian cancer patients receiving PARP inhibitors for different indications.

Indication for receiving PARP inhibitors	Number ( N = 54)	mPFS (months)
First-line maintenance	34	24
PSR (≥ 12 months)	13	NR
PSR (6–12 months)	7	9.6

PARP, poly(ADP-ribose) polymerase; PSR, platinum-sensitive recurrence; mPFS, median progression-free survival; NR,not reached.

**Statistical analysis:** Comparisons between Continuous variables’ groups were made using the t-test. Comparisons between Count variables’ groups were made using the chi-square or Fisher’s exact test. For multivariate analyses, Cox proportional hazard regression analyses performed to calculate hazard ratios (HRs) and 95% confidence intervals (CIs).

**Figure 2:**
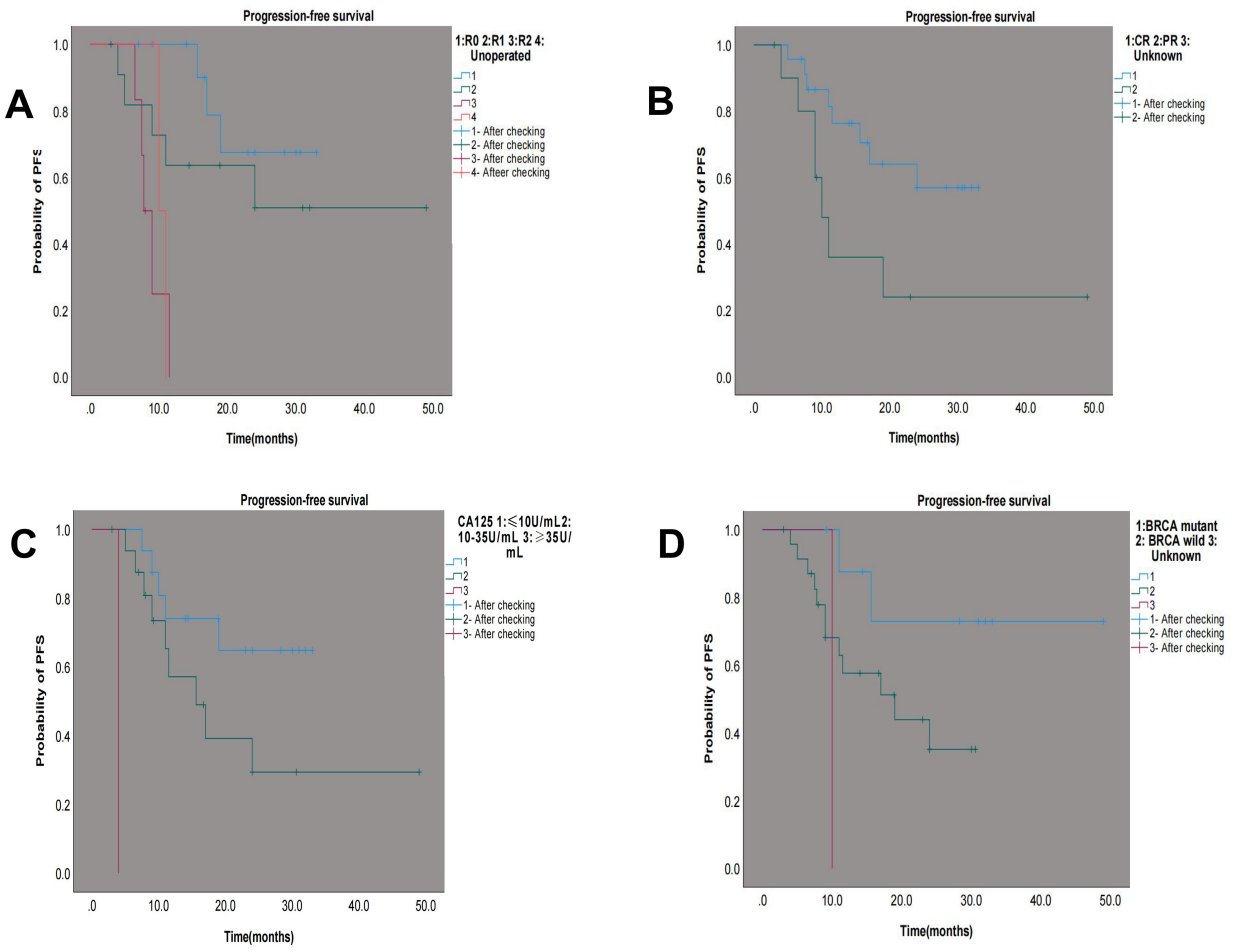
Factors associated with median progression-free survival (mPFS) in a cohort of patients with ovarian cancer. Effects of: **(A)** tumor cytoreduction status, **(B)** achieving CR or PR before receiving a PARP inhibitor treatment, **(C)** CA-125 level before receiving PARP inhibitor treatment, and **(D)** BRCA status, on mPFS.

Among the 34 first-line maintenance patients, 9 received olaparib and 25 received niraparib. mPFS was not reached in the olaparib subgroup and was 17.0 months in the niraparib subgroup. Univariate analysis identified four factors associated with mPFS: R0 resection at initial surgery, achievement of CR before PARP initiation, CA-125 level before PARP treatment (≤10, 10–35, or ≥35 U/mL), and BRCA mutation status (**Table 3**; **Figure 2**). Multivariate analysis confirmed R0 resection, BRCA mutation status, and pre-treatment CA-125 level as independent predictors of PFS. PARP inhibitor type, neoadjuvant chemotherapy, secondary cytoreductive surgery, International Federation of Gynecology and Obstetrics(FIGO) stage, and number of prior platinum regimens showed no significant effect on PFS.

**Table 3:** Factors associated with mPFS in older adult patients receiving PARP inhibitor as first-line maintenance therapy.

	Number (N = 34)	mPFS (months)	HR (95% CI)	Univariate analysis p	Multivariate analysis p
Type of PARP inhibitor received					
Olaparib	9	NR	3.613 (0.811-16.100)	0.072	
Niraparib	25	17.0			
Neoadjuvant chemotherapy					
Yes	10	19.0	1.429 (0.452-4.516)	0.541	
No	24	24. o			
Outcome of surgical treatment					
R0	13	NR	2.276 (1.330-3.896)	0.001*	0.004*
R1	12	NR			
R2	6	7.8			
inoperable	3	10.0			
Previous platinum-based chemotherapy					
≤6	16	24.0	1.215 (0.438-3.372)	0.708	
≥6	18	NR			
CA-125 level before PARP inhibitor treatment (U/mL)					
≤10	16	NR	3.463 (1.148-10.447)	0.019*	0.022*
10–35	17	15.6			
≥35	1	4			
Objective response to last platinum-based therapy					
CR	23	NR	2.883 (1.088-8.085)	0.036*	0.194
PR	11	10.0			
*BRCA* Mutation status					
Mutant type	9	NR	3.262 (1.045-10.189)	0.040*	0.022*
Wild-type	24	19			
Unknown	1	10			
FIGO stage					
II–III	26	24	1.324 (0.420-4.179)	0.631	
IV	8	17			

*Significant difference.

PARP, poly(ADP-ribose) polymerase; CR, complete response; PR, partial (PR) response; NR,not reached.

**Statistical analysis:** Comparisons between Continuous variables’ groups were made using the t-test. Comparisons between Count variables’ groups were made using the chi-square or Fisher’s exact test. For multivariate analyses, Cox proportional hazard regression analyses performed to calculate hazard ratios (HRs) and 95% confidence intervals (CIs).

### Safety and tolerability

Safety was evaluated in 22 patients who received olaparib and 36 who received niraparib (including the two switchers). Overall, 47 (83.9%) patients experienced any-grade adverse events (AEs). The most common AEs and grade 3–4 AEs for each drug are summarized in **Table 4**. Briefly, nausea was more frequent in the olaparib group (68.2% vs. 30.6%, p=0.007), and anemia was the most common grade 3–4 AE in the olaparib group (22.7%), whereas thrombocytopenia was most common in the niraparib group (11.1%). No significant difference in grade 3–4 AEs was observed between the two groups.

**Table 4:** Summary of adverse events.

	any grade			grade 3–4		
	Olaparib (22 cases))	Niraparib (36 cases)	P	Olaparib ( (22 cases))	Niraparib (36 cases)	P
Leucopenia	8 (36.4%)	6 (16.7%)	0.118	2 (9.1%)	2 (5.6%)	0.630
Thrombocytopenia	3 (13.6%)	10 (27.8%)	0.332	1 (4.5%)	4 (11.1%)	0.640
Anemic	11 (50.0%)	8 (22.2%)	0.044*	5 (22.7%)	3 (8.3%)	0.238
Nauseating	15 (68.2%)	11 (30.6%)	0.007*	2 (9.1%)	2 (5.6%)	0.630
Vomiting	2 (9.1%)	3 (8.3%)	1.000	2 (9.1%)	1 (2.8%)	0.551
Constipation	1 (4.5%)	5 (13.9%)	0.392	1 (4.5%)	0 (0.0%)	0.379
Stomach upset	2 (9.1%)	2 (5.6%)	0.630	0 (0.0%)	0 (0.0%)	1.000
Dysgeusia	0 (0.0%)	1 (2.8%)	1.000	0 (0.0%)	0 (0.0%)	1.000
Fatigue	4 (18.2%)	9 (25.0%)	0.747	0 (0.0%)	1 (3.0%)	1.000
Hypothyroidism	1 (4.5%)	3 (8.3%)	1.000	0 (0.0%)	0 (0.0%)	1.000
Renal insufficiency	0 (0.0%)	1 (2.8%)	1.000	0 (0.0%)	0 (0.0%)	1.000
Insomnia	0 (0.0%)	4 (11.1%)	0.287	0 (0.0%)	0 (0.0%)	1.000
Headaches	0 (0.0%)	1 (2.8%)	1.000	0 (0.0%)	0 (0.0%)	1.000
Numbness of the limbs	4 (18.2%)	1 (2.8%)	0.063	0 (0.0%)	0 (0.0%)	1.000
Joint pain	1 (4.5%)	0 (0.0%)	0.379	0 (0.0%)	0 (0.0%)	1.000
Palpitation	0 (0.0%)	4 (11.1%)	0.287	0 (0.0%)	0 (0.0%)	1.000
Hypertension	0 (0.0%)	2 (5.6%)	0.521	0 (0.0%)	0 (0.0%)	1.000

*Significant difference.

Olaparib group (n=22): includes 20 patients who received olaparib as initial therapy plus 2 patients who switched from olaparib to niraparib within 3 months (safety analysis based on olaparib exposure). Niraparib group (n=36): includes 34 patients who received niraparib as initial therapy plus the same 2 switchers (analyzed in the niraparib group for follow−up outcomes).

**Figure 3:**
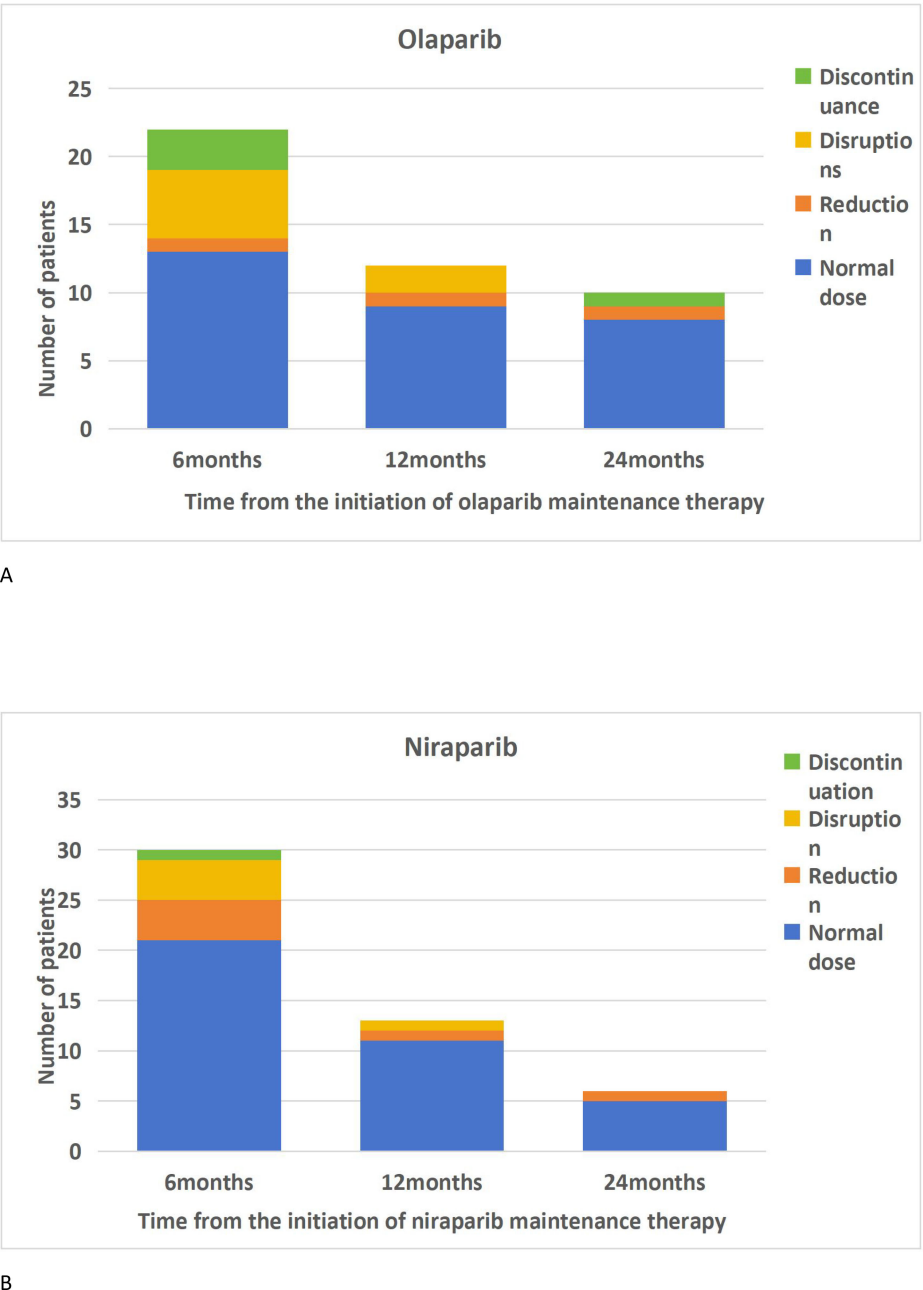
Tolerance to PARP inhibitors in the cohort included in this study. Dose adjustments at 6, 12, and 24 months in patients treated with: **(A)** olaparib and **(B)** niraparib. 4 patients who stopped receiving olaparib, 1 patient switched to niraparib therapy and 1 patient discontinued treatment 4 for intolerable grade IV vomiting, 1 case switched to niraparib for severe constipation and 1 case discontinued for the development of myelodysplastic syndrome (MDS) and died within 1 month. In the niraparib group, 4 cases had dose reductions, 9 cases had dose interruptions, and 1 case discontinued niraparib treatment due to a severe gastrointestinal reaction.

Dose reductions, interruptions, and treatment discontinuations are detailed in **Table 5**, **Figure 3**. Dose adjustments occurred predominantly within the first 6 months of treatment. The treatment termination rate was higher in the olaparib group than in the niraparib group (18.2% vs. 2.8%), although the difference was not statistically significant. One patient in the olaparib group discontinued after 13 months due to myelodysplastic syndrome (MDS) and subsequently died.

**Table 5:** Summary of dose adjustments for ovarian cancer patients in this study.

Dose adjustment	Olaparib	Niraparib	P
Reduction	3 (13.6%)	4 (11.1%)	1.000
Interruption	6 (27.3%)	9 (25.0%)	1.000
Termination	4 (18.2%)	1 (2.8%)	0.063

## Discussion and conclusions

### Summary of main results

This study is the first to evaluate the efficacy and safety of PARP inhibitors as first-line or PSR maintenance therapy in elderly patients with ovarian cancer.

### Results in the context of published literature

The mPFS in our study was 24 months, which was significantly longer than that of 8.2 months in the placebo group of newly-diagnosed ovarian cancer patients in the PRIME study (6). Furthermore, the mPFS for patients in the PSR group is a significant prolongation of the mPFS of 5.4 months reported for the placebo group in NORA (9). The mPFS of patients receiving niraparib as first-line maintenance therapy was 17 months, shorter than the results observed in the PRIME study (24.8 months) (6). This may be the proportion of patients with *BRCA*-mutant was relatively low among the elderly patients undergoing first-line maintenance therapy in our study (26.5% versus 36.7%) (9) and 8.8% (3/34) of patients were not treated surgically because of age and comorbidities.

Previous studies of olaparib administered as maintenance therapy in patients with platinum-sensitive recurrent *BRCA*-mutated ovarian cancer found that normalization of CA-125 and CR before receiving PARP inhibitors is associated with longer PFS (23, 24) and have highlighted a significant difference in the prognosis of patients with ovarian cancer with CA-125 ≤ 10 U/mL (25). Our study is the first to suggest that patients with CA125 ≤10 U/mL before initiating first-line maintenance therapy with PARP inhibitors will experience longer PFS.

*BRCA* mutations were found to impact mPFS of patients in first-line maintenance in this cohort, consistent with findings from the PRIME study (6). The *BRCA*1/2 mutation rate of 23.2% in patients with high-grade ovarian cancer in our study is consistent with that of 24.5% reported for patients with high-grade plasma cancers in previous studies (26) and it is lower than that reported by the PRIME study (36.7%) conducted in Chinese population (6). Our findings are consistent with previous randomized trials reporting that the mPFS of patients with *BRCA*-mutant tumors is significantly longer than that of those with wild-type *BRCA* cancer (8, 27).

Our study also found the most significant PFS benefit in elderly patients who achieved R0 in initial tumor cytoreduction, which is consistent with the recent meta-analysis demonstrating patients with no visible residual lesions treated with PARP inhibitors showed the most favorable PFS (28).

The incidences of AEs were generally lower than the incidence of grade 3 adverse reactions reported in the NORA phase 3 trial (9) and PRIME study (6). Similarly, our findings indicated lower rates of the most common grade 3–4 events reported in the PRIMA and NOVA studies (5, 8). The most common AE of any grade in the group receiving olaparib was nausea (68.2%) similar to the 68% reported in Study-19 (29). The most common grade 3–4 AE was anemia (22.7%), which is higher than the 5.1% reported in Study-19 (25), consistent with the 22% reported in SOLO1(4) and 21% reported in SOLO2 (30).

Dose reductions are effective in managing patient toxicity and PFS in patients receiving dose reductions of niraparib has been demonstrated to be similar to that of patients maintained on the original dose in the PRIMA trial (31). MDS is a severe complication associated with PARP inhibitors, with a median interval from initiation of PARP inhibitor treatment to MDS of 17.8 months, an incidence of approximately 1%–2%, and a mortality rate of around 45% (32). Regarding relapse, the final analysis of the SOLO2 study showed a higher rate (8%) of MDS with maintenance olaparib (30). In our study, 1 case (1.8%) of MDS occurred, consistent with the findings of the study. There is no significant difference in the AEs and risks of PARP inhibitors in elderly patients relative to the general population. The higher incidence of gastrointestinal reactions in elderly patients treated with olaparib than those receiving niraparib is consistent with the results of the population-wide randomized clinical trial (8, 29).

### Strengths and weaknesses

Although prior subgroup analyses of pivotal trials and several real-world studies have provided valuable data on PARP inhibitor use in elderly patients with ovarian cancer (4, 5, 7, 8), evidence specifically focused on both first-line and platinum-sensitive recurrent (PSR) maintenance settings in a single real-world elderly cohort remains limited. Our study therefore adds to the existing literature by describing the efficacy, safety, and prognostic factors in this understudied population.It has limitations, including poor generalizability of the data because of the single-center study design, and selection bias attributable to the retrospective analysis. Our study is limited by the small sample size (n=56), which leads to reduced statistical power, particularly after subgroup stratification by treatment line or drug type. Consequently, non-significant subgroup comparisons may be false negatives, and the precision of PFS estimates (especially in small subgroups, e.g., n=7 for PSR 6–12 months) is limited. The multivariate analysis was constrained to three covariates to avoid overfitting. Therefore, our findings should be considered exploratory and hypothesis-generating, requiring confirmation in larger prospective studies. The follow-up period needs to be longer to achieve mPFS in some subgroups and determine overall survival outcomes. Five patients (8.9%) in the cohort were not tested for BRCA mutations, which may affect the interpretation of the findings to some extent. Another consideration is the definition of “elderly.” Although we adopted the commonly accepted cutoff of age ≥65 years based on NCCN guidelines and Chinese expert consensus (18, 19), it is important to recognize that chronological age alone does not fully capture physiological and functional heterogeneity among older adults. Future studies should incorporate geriatric assessment tools, such as the G8 screening tool or comprehensive geriatric assessment (CGA), to better evaluate treatment tolerability and outcomes in this population (19).

### Implications for practice and future research

It is evident that maintenance therapy with olaparib and niraparib is effective in elderly patients with ovarian cancer. Chronological age alone should not preclude PARP inhibitor therapy (33). Normalization of CA-125 levels and R0 resection at initial surgery are key treatment goals that should be actively pursued. Although this retrospective study did not incorporate formal geriatric assessment (GA) tools, current guidelines—including the American Society of Clinical Oncology(ASCO) Global Guideline on Geriatric Assessment (2025) and International Society of Geriatric Oncology(SIOG) recommendations—strongly endorse the use of screening tools such as G8 and VES-13 for older patients with cancer, followed by comprehensive geriatric assessment (CGA) when abnormalities are detected (34, 35). Most dose modifications occur within the first 6 months, warranting close monitoring during this period. Prospective studies incorporating validated GA tools are needed to optimize treatment individualization in elderly ovarian cancer patients.

### Conclusion

Our data demonstrates that maintenance therapy with olaparib and niraparib is effective and well-tolerated in real-world clinical practice in Chinese elderly ovarian cancer patients. Three factors were identified as predictors of longer PFS in older patients with ovarian cancer receiving PARP inhibitors as first-line maintenance therapy: initial surgery to R0, *BRCA* mutant type, and CA-125 ≤10 U/mL before receiving PARP inhibitors.

## Data Availability

The raw data supporting the conclusions of this article will be made available by the corresponding author, without undue reservation, to any qualified researcher.
